# Surgical approach for kidney transplantation under spinal anesthesia

**DOI:** 10.1093/jscr/rjaa538

**Published:** 2020-12-31

**Authors:** Atta Nawabi, Peter Sullivan, Martin De Ruyter, Amy Pichoff, Clay D King, Perwaiz Nawabi

**Affiliations:** Department of Surgery, The University of Kansas, Kansas City, KS, USA; Department of Surgery, The University of Kansas, Kansas City, KS, USA; The Kansas City University, Kansas City, MO, USA; The Kansas City University, Kansas City, MO, USA; Department of Anesthesia, The University of Kansas, Kansas City, KS, USA; Department of Anesthesia, The University of Kansas, Kansas City, KS, USA

## Abstract

Kidney transplantation is the treatment of choice for patients with end-stage renal disease (ESRD). It has been shown to improve quality of life as well as extending life of patients with ESRD as compared to renal replacement therapy (5-year survival rate of 68% after transplant vs 36% dialysis) (Hart A, Smith JM, Skeans MA. OPTN/SRTR 2015 annual data report: kidney. Am J Transplant 2017;17:21–116). Traditionally, patients undergo general endotracheal tube anesthesia for this surgery. During the COVID-19 pandemic, general anesthesia drugs and airway equipment were in short supply. Additionally, airway manipulation was avoided when possible due to concern for virus spread from aerosolizing procedures (i.e. intubation/extubation). In this case report, we review a 65-year-old female with an ESRD due to hypertension and diabetes that underwent deceased donor kidney transplant under spinal anesthesia. We will further discuss the benefits of spinal anesthesia in renal transplant operations.

## INTRODUCTION

Anesthesia allows patients to undergo surgical or interventional procedures by producing absence of awareness or anxiety, analgesia, amnesia, and adequate muscle relaxation or immobility. There are multiple types of anesthesia consisting of general anesthesia, regional anesthesia (peripheral nerve blocks), local anesthesia and monitored anesthetic care (sedation).

General anesthesia is most used for major surgical procedures. It is induced with intravenous medications or medications inhaled through a mask. Once patients become unconscious, an endotracheal tube or a supraglottic airway (i.e. laryngeal mask airway) is placed. General anesthesia is maintained during surgery by intravenous medications or anesthesia gases.

Spinal anesthesia is a form of regional anesthesia in which local anesthetic is placed into the subarachnoid space via a needle or catheter, which results in numbness to a large portion of the body, often T4 dermatome and below. This form of anesthesia can be utilized for lower abdominal, pelvic and lower extremity operations.

## CASE REPORT

A 65-year-old female with a history of insulin-dependent diabetes, hypertension, congestive heart failure, hypothyroidism and end-stage renal disease (ESRD) on hemodialysis presents for diseased donor kidney transplant. Her preoperative vitals were stable, and her starting hemoglobin was 12.1 grams/deciliter with a platelet count of 181 000. Her last dialysis session was the day before transplant. After proper inspection of the donor kidney, she was taken to the operating room for kidney transplant. She was given 1 mg of midazolam and 50 mcg of fentanyl for sedation prior to the procedure. A single shot spinal was performed utilizing a midline approach at the level between L2-3 with a 27-gage, pencil tip spinal needle. 1.8 mL of bupivacaine PF 0.75% was placed in the intrathecal space with a sensory block up to the T4 dermatome. The patient was given a low dose (20–60 mcg/kg/min) propofol infusion for sedation during the transplantation. The patient then underwent a kidney transplant through a right lower quadrant curvilinear incision utilizing standard transplant surgical technique. At the conclusion of the case, sedation was discontinued, and patient was taken to the recovery room.

## DISCUSSION

To this date, there is only one other case report on spinal anesthesia for kidney transplants [1]. In the past, neuraxial anesthesia has been avoided due to the concern for ESRD induced coagulopathy [2]. Adequate coagulation is important when performing neuraxial anesthesia procedures to decrease the risk of epidural hematoma. Patients that have an INR >1.5, aPTT >35 s and platelets <100 k are at increased risk of epidural hematoma [3]. There are no definitive studies looking at the safety of spinal or epidural procedures in patients with ESRD with no clinical or laboratory abnormalities that would indicate compromised coagulation that would increase the risk of epidural hematoma. The risk of epidural hematoma with a single shot spinal is theoretically lower due to the smaller gage need used and the absence of catheter shearing on placement and removal. However, there have been reports of epidural successful epidurals in end-stage renal patients [4]. A benefit to performing renal transplant under spinal anesthesia is the increased pain control in the immediate postoperative period.

In conclusion, this case illustrates renal transplantation can successfully be done under spinal anesthesia. Further investigation assessing safety, pain control, and hospital course would be worthwhile.
